# Beyond the hype: re-evaluating efficacy metrics and modeling rigor for MSC-EVs-based therapy in acute brain injury

**DOI:** 10.3389/fmed.2025.1654429

**Published:** 2025-12-04

**Authors:** Fating Zhou, Hongxia Wang, Xiaodan Zhu, Rui Huang, Xuemei Jiang, Haizhen Duan, Yu Ma, Shanmu Ai

**Affiliations:** 1 Emergency Department, Chongqing Emergency Medical Center, Chongqing University Central Hospital, Bioengineering College, Chongqing University, Chongqing, China; 2 Chongqing Key Laboratory of Emergency Medicine, Chongqing, China; 3 Department of General Practice, People’s Hospital of Deyang, Deyang, Sichuan, China; 4 Department of Emergency Medicine, Affiliated Hospital of Zunyi Medical University, Zunyi, Guizhou, China

**Keywords:** extracellular vesicle, mesenchymal stem cell, ischemic-reperfusion injury, cardiac arrest, traumatic brain injury, hypoxic brain injury, miRNA

## Abstract

Acute brain injuries (ABI), such as traumatic brain injury, stroke, hypoxia-induced brain injury, and cardiac arrest, are critical and life-threatening conditions that contribute to substantial mortality and long-term disability. Despite extensive translational efforts, no effective therapy has improved long-term functional outcomes, highlighting a critical unmet need. Mesenchymal stem cell-derived extracellular vesicles (MSC-EVs) have emerged as promising cell-free therapeutic platform, offering multifaceted repair capabilities. This review synthesizes current evidence supporting the neuroprotective effects of MSC-EVs, which operate through synchronized immunomodulation, anti-apoptotic signaling, enhancement of neurogenesis, and stimulation of angiogenesis. We further delineated the fundamental EVs biology, including biogenesis pathways, spatiotemporal biodistribution, and blood–brain barrier (BBB) trafficking mechanisms that underpin therapeutic efficacy. Collectively, we established MSC-EV cargo as a strategic solution to unmet neuroprotective needs while mapping clinical translation roadmaps to accelerate the rational development of regenerative neurotherapeutics.

## 1 Introduction

Acute brain injury (ABI) is a common syndrome with poor prognosis and high disability in the emergency department and intensive care unit (1, 2). This condition encompasses diverse etiologies, including ischemic stroke (IS), traumatic brain injury (TBI), neonatal hypoxic-ischemic encephalopathy (HIE), and cardiac arrest (CA), which share common pathophysiological pathways such as excitotoxicity, neuroinflammation, and blood–brain barrier (BBB) disruption (3). Preclinical studies have demonstrated the efficacy of several drugs for mitigating ABI and ameliorating neurological deficits in animal models (4–6). However, these findings have largely failed to translate into successful clinical outcomes. Consequently, the development of novel therapeutic strategies for ABI is imperative.

Accumulating evidence has revealed that mesenchymal stem cells (MSCs) and their extracellular vesicles (MSC-EVs) exert therapeutic effects in ABI through multiple mechanisms. These include (1) secretion of neurotrophic factors (e.g., nerve growth factor, epidermal growth factor, and brain-derived neurotrophic factor), (2) inhibition of microglial activation and neuroinflammation, (3) suppression of neuronal apoptosis, and (4) promotion of synaptic remodeling (7, 8). Compared with parental MSCs, MSC-EVs are promising therapeutic candidates for ABI because of their superior BBB penetrability, modifiable membrane properties, enhanced stability, and favorable storage profiles.

Preclinical studies in animal models of middle cerebral artery occlusion (MCAO), TBI, and hypoxic-ischemic brain damage (HIBD) have demonstrated that MSCs and their EVs improve cognitive and motor deficits, correlating with microglial deactivation and reduced release of inflammatory factors (9–13). Proteomic analyses of BMSC-EVs have identified a protein cargo exceeding 700 distinct molecules that are significantly enriched in immune regulation and angiogenic pathways (14). Furthermore, MSC-EVs transport multifaceted bioactive substances, including non-coding RNAs (miRNAs and lncRNAs), genomic DNA fragments, and phospholipid mediators, which orchestrate critical pathophysiological processes, including cellular proliferation, programmed apoptosis, and autophagic flux modulation (15–17). Given their neurorestorative potential in ABI models, MSC-EVs are promising therapeutic candidates. Supporting this, Zhang et al. (18) and Lv et al. (19) collectively demonstrated that MSC-EVs reduced lesion volume and enhanced neurological function in MCAO models, primarily by attenuating neuronal apoptosis and promoting axonal growth.

Despite supporting evidence from multiple studies (Table 1; Supplementary Tables S1, S2), significant heterogeneity exists regarding the optimal EV dosage, administration route, frequency, and timing across preclinical models (18–54). Key translational challenges further limit efficacy: (1) rapid systemic clearance of intravenously administered EVs by macrophages and neutrophils; (2) the BBB acting as a physiological barrier restricting peripheral EV entry into the CNS; and (3) insufficient intrinsic bioactivity and scalable production yields of native EVs, necessitating bioengineering enhancement. Therefore, the therapeutic efficacy of MSC-EVs in preventing ABI remains controversial. To address these limitations, this review comprehensively analyzes the EV biogenesis pathways, systemic biodistribution kinetics, and engineered BBB traversal strategies that leverage receptor-mediated transcytosis. Additionally, we synthesized findings on the therapeutic potential of distinct MSC-EV subtypes in ameliorating non-infectious acute brain injuries. The biogenic pathways of EVs primarily determine their subtype properties through cargo sorting mechanisms and membrane composition, significantly limiting the therapeutic efficacy of EV-based ABI targeting.

**Table 1:** MSC-EVs alleviated ischemia stroke *in vivo*.

Species	Cells	Administration route	Time	Dose of EVs	References
C57BL	BMSC	Tail vein	After MCAO	NA	(20)
C57BL	BMSC	Tail vein	After MCAO	Released by 2 × 10^6^ MSCs	(21)
C57BL	BMSC	Tail vein	90 min after MCAO	10^10^	(22)
C57BL	BMSC	Tail vein	2 h after reperfusion	200 μL	(23)
C57BL	BMSC	Tail vein	24 h after MCAO	200 μg	(24)
C57BL	BMSC	Tail vein	12 h after reperfusion	300 μg	(25)
SD rat	BMSC	Tail vein	The next day of MCAO and 14 day later	200 μL	(26)
SD rat	BMSC	Tail vein	10 min after MCAO	100 μg	(27)
SD rat	BMSC	Lateral ventricle	24 h after MCAO	100 μg	(28)
Postnatal day 9–10 C57BL	BMSC	Lateral ventricle/intranasal	At the time of reperfusion	1 μg/μL or 5 μg/μL	(29)
Rat pups	UC-MSC	Tail vein	After MCAO	150 μg	(30)
SD rat	UC-MSC	Tail vein	After MCAO	100 μg/day for 3 days	(31)
SD rat	ADMSC	Lateral ventricle	Before MCAO	100 μg/kg/day for 4 days	(18)
SD rat	ADMSC	Lateral cerebral ventricle	Before MCAO	100 μg/kg/day for 3 days	(32)
SD rat	ADMSC	Tail vein	1 h after MCAO	150 μg	(19)
SD rat	NSC	Lateral ventricle	2 h after surgery	30 μg	(33)
SD rat	NSC	Tail vein	After 1 h of MCAO	300 μg	(34)

ADMSC, adipose mesenchymal stem cell; BMSC, bone marrow mesenchymal stem cell; MCAO, middle cerebral artery occlusion; NSC, nerve stem cell; UC-MSC, umbilical cord mesenchymal stem cell.

## 2 Biogenesis of EVs

EVs are lipid bilayer-enclosed nanoparticles that are constitutively secreted by all nucleated cells and serve as key mediators of intercellular communication (55). Based on their biogenic mechanisms and physical properties, EVs are operationally classified into three primary subtypes: (1) exosomes (30–150 nm) originating from endosomal multivesicular bodies; (2) microvesicles (MVs, 50–1,000 nm) generated via plasma membrane budding; and (3) apoptotic bodies (500–2,000 nm) released during programmed cell death (56). EVs with diameters <200 nm is commonly designated as “small EVs” (sEV). Critically, the Minimal Information for Studies of Extracellular Vesicles (MISEV2018) guidelines advocated using the size-based term “small EVs” (sEVs) over biogenesis-based terms like “exosomes” or “microvesicles,” provided size is rigorously determined (57).

Beyond size differences, the three subtypes of EVs originated through distinct biogenesis pathways. Exosome formation commences with the plasma membrane invagination, which generates early endosomes. These endosomes recruit the endosomal sorting complex required for transport (ESCRT) machinery to mediate inward budding, culminating in multivesicular body (MVB) maturation (58). Subsequent MVB docking and fusion with the plasma membrane release intraluminal vesicles into the extracellular space as exosomes (59). Conversely, microvesicles are formed by direct outward budding and fission of the plasma membrane, whereas apoptotic bodies arise from programmed membrane blebbing during cellular apoptosis (59). Compositional profiling of EVs via transmission electron microscopy and western blot revealed enrichment of the characteristic components, including sphingomyelin, cholesterol, phosphatidylserine, tetraspanins (CD9, CD63, and CD81), and heat shock proteins (HSP70 and HSP90). Furthermore, EVs encapsulate diverse donor cell-derived cargos, including nucleic acids (genomic DNA, mRNA, miRNA, and siRNA) and functional proteins (60). These bioactive payloads, particularly miRNAs, mediate the cross-cellular regulation of cell proliferation and apoptosis through recipient cell internalization via endocytic pathways.

In the pathophysiology of ABI, EVs secreted by bone marrow-derived mesenchymal stem cells (BMSCs), adipose-derived mesenchymal stem cells (ADMSCs), and neural progenitor cells (NPCs) mediate neuroprotection through two mechanisms: (1) inhibition of caspase-3-dependent apoptotic pathways and (2) attenuation of reactive oxygen species (ROS)-induced oxidative stress. This concerted action promoted neural circuit repair (61, 62). Similar to the role of biogenic pathways, the *in vivo* distribution of EVs, particularly their accumulation in the brain, critically governs the efficacy of EV-based therapeutics for ABI. Subsequently, we delineated the systemic distribution patterns of EVs and the mechanisms underlying their traversal across the BBB.

## 3 Biodistribution of BMSC-EVs

The therapeutic efficacy of peripherally administered MSC-EVs requires efficient biodistribution to the cerebral parenchyma, necessitating the optimization of intracranial delivery strategies (63). EVs biodistribution is route dependent and is influenced by parental cell tropism and surface molecular signatures (63). Intravenous administration triggers rapid clearance by the mononuclear phagocyte system, with residual vesicles predominantly accumulating in hepatic Kupffer cells, renal proximal tubules, and splenic macrophages (64, 65). Comparatively, intranasal delivery achieved significantly higher brain EV concentrations via the olfactory ependymal bypass pathway (64). Crucially, EVs distribution correlated with cellular origin; MSC-EVs were localized primarily to the liver, lungs, and spleen, whereas microglia-derived EVs showed abundant hepatic and cerebral accumulation (66). Consistently, NSC-EVs demonstrated superior intracranial distribution over BMSC-EVs in MCAO models (67).

The EVs membrane surface displayed functionally critical transmembrane proteins, lipids, and glycans, predominantly featuring tetraspanins (CD9/CD63/CD81), integrins (α4β1/α5β1), and major histocompatibility complexes. Notably, EVs co-expressing quadruple transmembrane proteins and integrin α4 exhibit enhanced tropism toward endothelial cells (68). Phosphatidylglycines and polysaccharides concurrently modulate cellular uptake of MSC-EVs (69). These findings establish a rationale for achieving intracranial EV targeting through engineered modifications of surface molecules via chemical conjugation or genetic engineering (70). Critically, the biodistribution of MSC-EVs is correlated with their pathophysiological state. In a comparative study of AKI and healthy mice, intravenous MSC-EVs showed accelerated renal accumulation in an AKI cohort (70). Similarly, macrophage-derived EVs demonstrated a 3-fold increase in BBB transmigration during intracranial inflammation compared to that under physiological conditions (71).

## 4 Mechanism of transport of MSC-EVs across the blood–brain barrier

The synthesis and biological distribution of MSC-EVs have been described previously. In this section, we analyze the mechanism of MSC-EV transport across the BBB. Evidence indicates that peripherally administered MSC-EVs must traverse the BBB to exert neuroprotective effects. Central nerve markers, including *α*-synuclein and microtubule-associated proteins, have been detected in EVs derived from peripheral organs and blood under physiological and pathological conditions (72, 73). Furthermore, EVs have been established as mediators of CNS-peripheral communication (74). However, the mechanisms underlying EV transport across the BBB remain unclear.

The BBB, a selective interface between systemic circulation and CNS, dynamically regulates molecular exchange to maintain homeostasis and excludes neurotoxic agents (75). This constitutes the primary obstacle to the development of CNS-targeted therapeutics. Under physiological conditions, the BBB selectively allows several small substances, such as lipid- and water-soluble small molecules, to enter the brain tissue (76). However, molecules greater than 1 KD cannot cross this barrier (75). A minority of large molecules, such as carbohydrates and essential amino acids, can cross the BBB via transporter proteins and receptors on the surface of endothelial cells (76). Hydrophilic molecules, such as hormones and lipoproteins, can cross the BBB via transcytosis (77).

**Figure 1:**
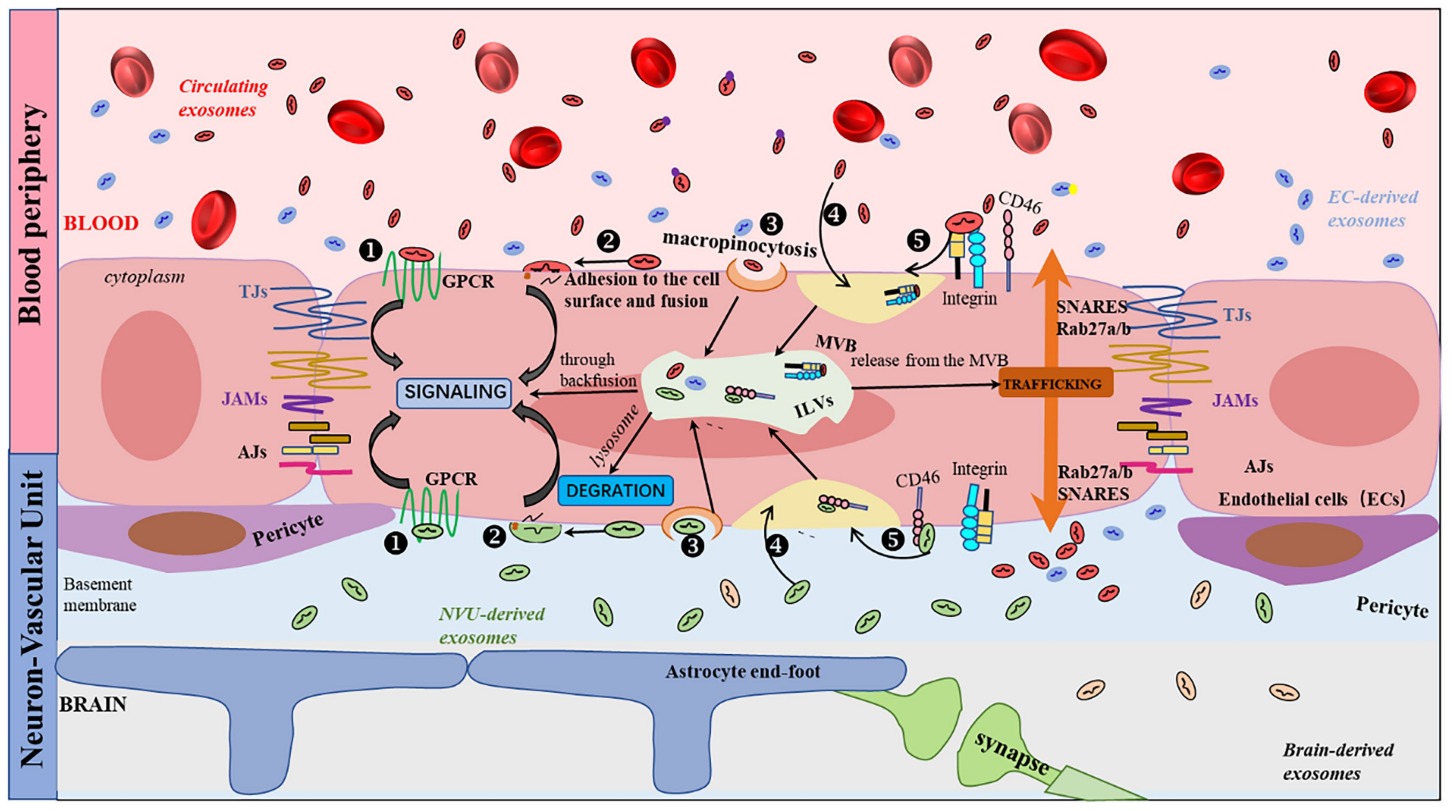
Schematic representation of the EVs transport pathway across the BBB. Five routes have been described for the interaction of EVs with receiving cells: (1) binding to protein G-coupled receptors on the cell surface, leading to the induction of signaling cascades; (2) adhesion and fusion to the cell surface, releasing the cytoplasmic content of EVs, which can result in a variety of events, including cellular signaling; (3) macropinocytosis; (4) nonspecific/lipid rafts; and (5) receptor-mediated transcytosis. There are three common outcomes of EVs: (i) degradation by lysosomes, (ii) induction (87) of signaling by releasing their contents into the cytoplasm through back-fusion events of the MVB, or (iii) translocation from the MVB to the plasma membrane as neoformed ILVs in the recipient cell.

Currently, the mechanism by which EVs cross the BBB remains to be fully understood; however, five theoretical routes have been suggested: G protein-coupled receptor-mediated transport, macropinocytosis, transcytosis, lipid rafts, and receptor-mediated transcytosis (Figure 1). Among these, clathrin-mediated transcytosis is crucial for receptor-mediated transcytosis (74). When ligands bind to receptors, ligand-receptor compounds are concentrated in clathrin-coated pits created by clathrin and adaptor proteins. Once clathrin-coated pits are separated from the plasma membrane, the vesicles lose their clathrin coat and fuse with early endosomes. Finally, the cargo is sorted and released on the opposite sides of the cell membrane (78). In a BBB model, Zhao et al. (79) identified that HEK 293-derived EVs cross the BBB via receptor-mediated endocytosis, lipid rafts, and macropinocytosis. In contrast, Terasaki et al. (80) revealed that EVs transport across the BBB was highly linked to integrins and CD46 on the endothelial cell surface, and that the number of EVs crossing the BBB decreased 2-fold after CD46 knockdown. Upon entering the cerebral microvascular endothelium, most EVs bind to lysosomes and are rapidly degraded; some fuse inversely with MVB and release their contents into the cytoplasm. The remaining EVs fuse with the plasma membrane via the MVB to form new ILVs (Figure 1).

## 5 MSC-EVs in ABI

### 5.1 Ischemic stroke and MSC-EVs

Ischemic stroke is one of the leading causes of death worldwide, and approximately 75% of survivors suffer from disabilities (81). Current FDA-approved therapies, such as recombinant tissue-type plasminogen activator (rt-PA) and endovascular thrombectomy, remain limited by narrow therapeutic windows and stringent eligibility criteria (82, 83). Therefore, novel strategies are urgently required to mitigate I/R injury and improve neurological outcomes. Stem cell-derived EVs have significant neuroprotective effects in ischemic stroke models. BMSC-EVs are the most extensively studied subtype, followed by adipose mesenchymal stem cell-derived EVs (ADMSC-EVs) and umbilical cord-derived MSC-EVs (UCMSC-EVs) (60).

**Figure 2:**
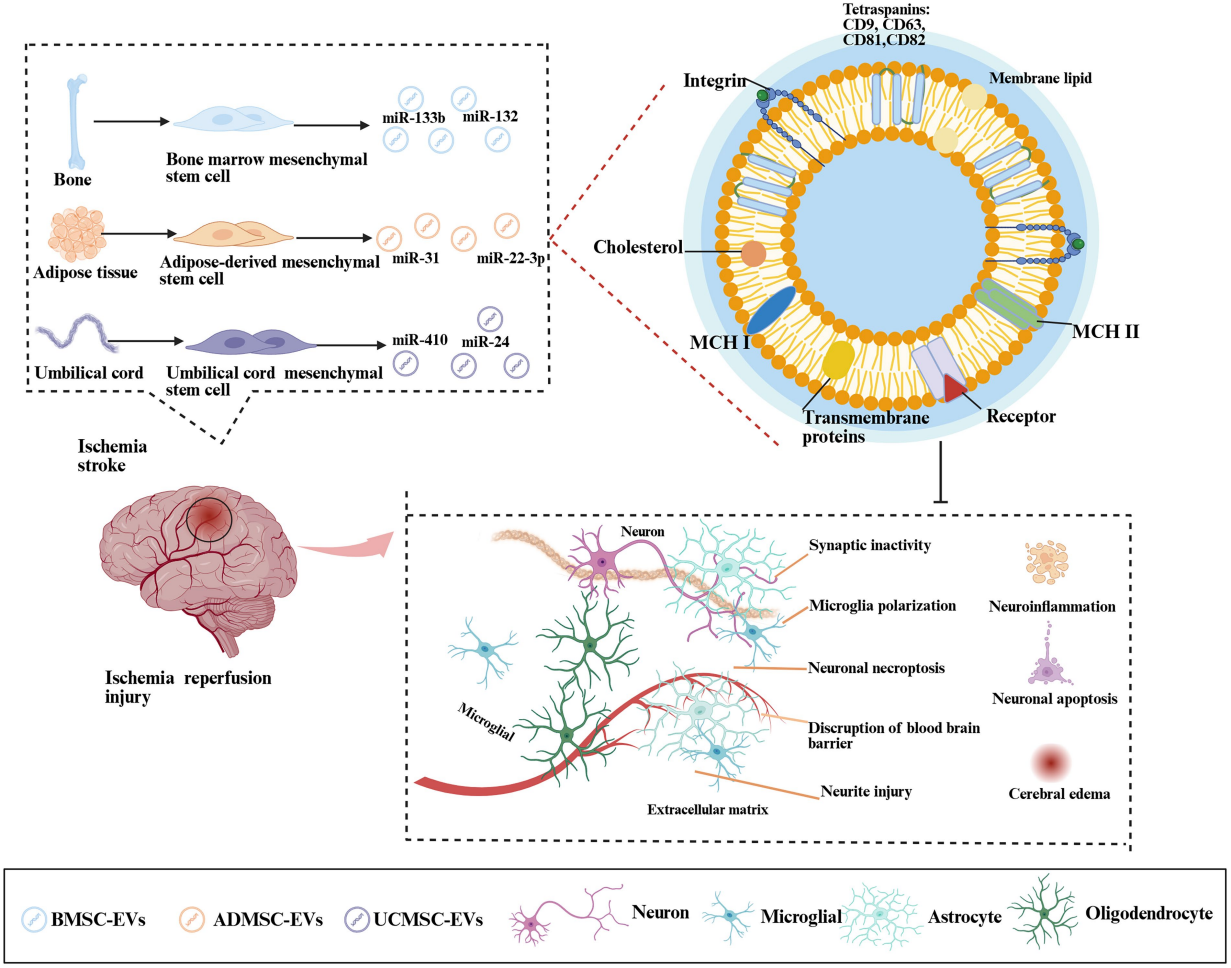
Mechanism by which MSC-EVs regulate cerebral ischemia-reperfusion injury. Ischemic stroke triggered cerebral hypoxia-ischemia, driving pathological cascades—metabolic dysfunction, cellular edema, microglial polarization, neuroinflammation, blood–brain barrier (BBB) disruption, and synaptic damage. BMSCs, ADMSCs, and UCMSCs secreted EVs that delivered miRNAs (miR-133, miR-132, and miR-31) to mitigate neuroinflammation, inhibit neuronal apoptosis, reduce edema, and promote cognitive recovery.

BMSC-EVs alleviate post-ischemic neuronal damage via various mechanisms, including immunomodulation, anti-apoptosis, inhibition of autophagy and oxidative stress, promotion of neuronal proliferation, and BBB improvement (Figure 2). These effects were mediated through key pathways: AMPK/mTOR, ACVR2B/p-Smad2/c-Jun, and JAK/AKT/GSK-3β/Wnt3 (21, 30, 84). A recent study confirmed that BMSC-EVs suppress neuronal apoptosis, decrease lactate dehydrogenase release, and promote neuronal proliferation after stimulation with oxygen-glucose deprivation/reoxygenation (84). Similarly, Feng et al. (24) revealed that BMSC-EV-derived miR-132 inhibits neuronal apoptosis via the Acvr2b/*p*-Smad 2/c-jun pathway (Figure 2).

In the MCAO model, BMSC-EVs-derived-miR-133b attenuated neuronal injury by targeting JAK1, thereby suppressing the release of inflammatory factors (85). Similarly, miR-132-3p enrichment in BMSC-EVs achieved via donor cell overexpression activates the Ras/PI3K/p-Akt/eNOS pathway, enhancing BBB integrity and cerebral perfusion (22). Parallel engineering approaches generated miR-26a-5p-enriched BMSC-EVs that inhibited CDK6 expression and microglial apoptosis (86), as well as miR-223-3p-modified BMSC-EVs that suppressed M1 microglial polarization, reduced pro-inflammatory cytokines, diminished infarct volume, and improved neurological scores (26).

ADMSC-EVs demonstrated comparable neuroprotective efficacy by reducing neuronal apoptosis, autophagy, and infarct volume (18). Zhang et al. (18) showed that ADMSC-EV-derived miR-22-3p targets KDM6B, which inhibits neuronal apoptosis via the BMP/BMF signaling pathway. Lv et al. (19) reported that the miR-31-mediated blockade of the TRAF6/IRF5 axis improved post-injury motor function. Notably, genetically engineered PEDF-ADMSC-EVs exhibit significantly enhanced anti-apoptotic activity relative to their unmodified counterparts (32), inhibiting neuronal apoptosis more efficiently than conventional approaches.

Umbilical cord MSC-derived EVs (UC-MSC EVs) modulate neuroinflammation by suppressing M1 glial polarization and attenuating inflammatory responses (87). These vesicles delivered miR-24 to downregulate AQP4 expression and activate the p38 MAPK/ERK/PI3K/AKT pathway, thereby collectively ameliorating ischemia-reperfusion-induced neuronal apoptosis (87).

### 5.2 Traumatic brain injury and MSC-EVs

Traumatic brain injury (TBI), a serious global public health problem, is a frequent and severe neurological illness encountered in emergency medicine (88, 89). Annually, more than 27 million cases of TBI are reported worldwide. TBI survivors frequently experience persistent cognitive, motor, and memory deficits, which are the leading causes of mortality and disability in adults under 45 years of age (88). Pathophysiologically, primary mechanical insults induce cerebral hemorrhage and tissue edema, whereas secondary injury cascades trigger excitotoxicity, mitochondrial dysfunction, neuroinflammation, axonal degeneration, and apoptosis (17). Current clinical management stratifies patients by severity: patients with mild to moderate TBI receive medical interventions, including intracranial pressure control, seizure prophylaxis, and targeted temperature management, whereas those with intracranial hematomas or severe contusions require surgical decompression (88, 89). Although these approaches mitigate acute symptoms and preserve vital function, they fail to address the underlying pathomechanisms, leaving long-term recovery contingent on endogenous repair processes. Emerging preclinical evidence has demonstrated that BMSC-EVs and ADMSC-EVs suppress post-TBI neuroinflammation and significantly improve functional recovery metrics in animal models (90, 91).

**Figure 3:**
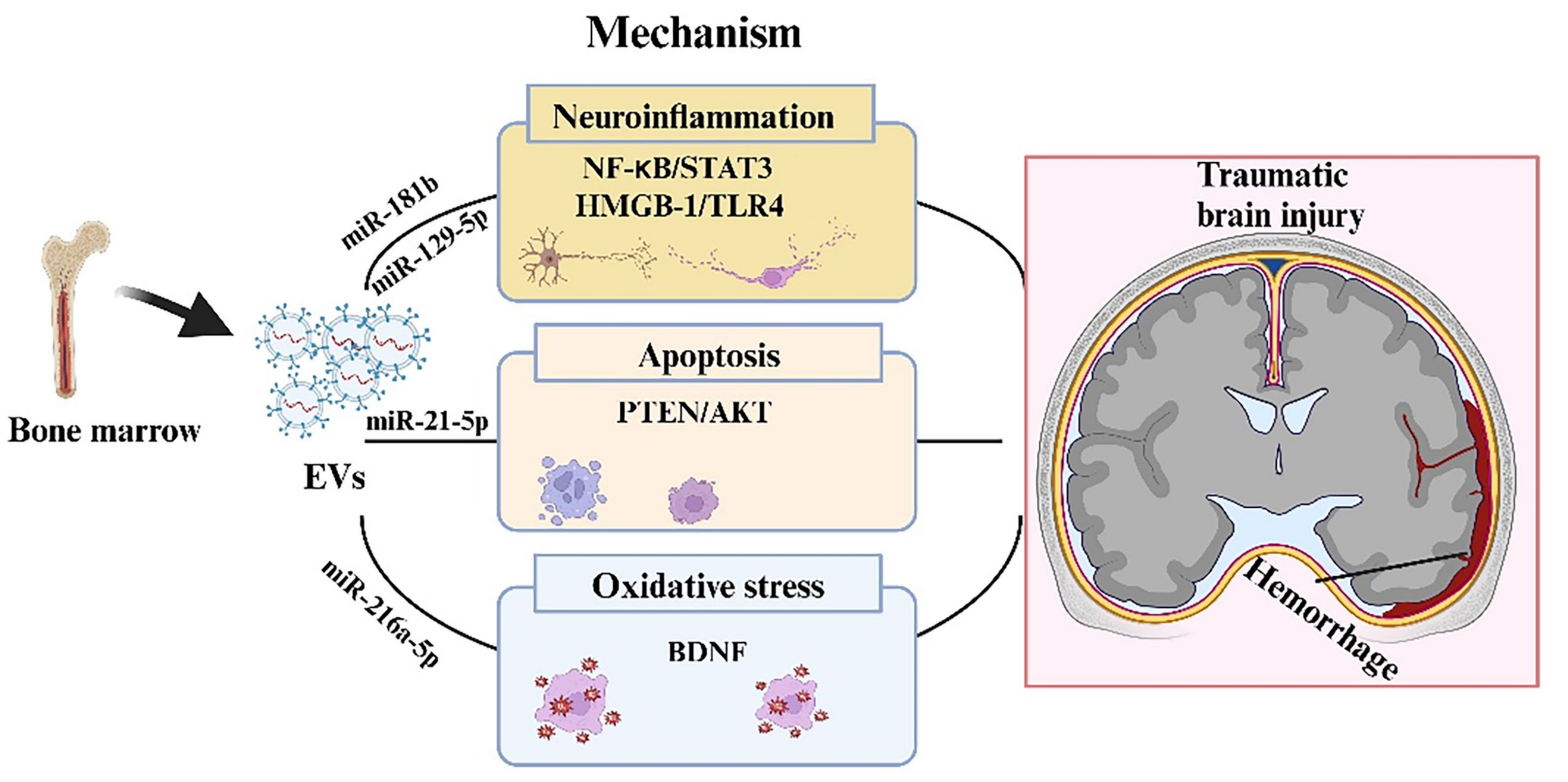
Mechanism of BMSC-EV regulation of traumatic brain injury. In the process of traumatic brain injury, BMSC-EVs during traumatic brain injury, BMSC-EVs transported a variety of miRNAs, which can suppress the expression of target genes and regulate neuroinflammation, apoptosis, and oxidative stress, involving several signaling pathways such as NF-kB/STAT 3, HMGB-1/TLR4 and PTEN/AKT.

BMSC-EVs effectively improve brain injury after TBI through multifaceted mechanisms, including regulating microglial activation, reducing neuroinflammatory factors and oxidative stress responses, improving cerebral perfusion, and promoting angiogenesis (Figure 3) (35, 92). These effects are primarily mediated by EV-encapsulated miRNAs. The miR-181b/STAT3 axis is a key regulator of neuroinflammation, with BMSC-EVs suppressing NF-κB activation to mitigate post-TBI inflammatory responses (37). Parallel findings revealed that miR-216a-5p from BMSC-EVs enhanced neuroplasticity by modulating BDNF-dependent mechanisms, significantly improving spatial learning in TBI models through the coordinated regulation of cell migration and apoptosis (43). Complementary studies have demonstrated that the miR-17-92 cluster confers hippocampal neuroprotection, preserving dentate gyrus integrity, while stimulating neovascularization and neurological recovery (42).

In addition to their direct neuronal effects, BMSC-EVs exhibit pronounced cerebrovascular benefits. While failing to modulate systemic hemodynamics in porcine TBI models, they significantly reduced intracranial pressure while enhancing cerebral perfusion (93). This vascular modulation was extended to subarachnoid hemorrhage models, where miR-21-5p-enriched BMSC-EVs ameliorated cerebral edema and cognitive deficits through PTEN/AKT pathway inhibition (39). Notably, similar neuroprotection was achieved via the miR-129-5p-mediated suppression of HMGB1/TLR4 signaling (38). suggesting conserved mechanisms across injury models (Figure 3). Collective evidence suggests that BMSC-EVs are multimodal therapeutic agents capable of simultaneously targeting neuroinflammation, vascular dysfunction, and excitotoxicity with particular efficacy against glutamate-mediated neurotoxicity and p38 MAPK activation (36). These findings underscore the translational potential of EV-based interventions in complex TBI pathophysiology.

Apart from BMSC EVs, ADMSC-EVs also repressed microglia and macrophage cell activity, relieved TBI impairment by suppressing the NF-κB and MAPK pathways (44). Intracardial injection of UC-MSC EVs into neonatal rats with ventricular hemorrhage significantly improved the motor coordination of injured rats (94). UC-MSC EVs also attenuated inflammation and apoptosis, while the neuroprotection can be reversed when BDNF expression was downregulated (94). Notably, Li et al. (95) found that exfoliated deciduous teeth cell-derived EVs inhibited the release of inflammatory factors and reduced cortical lesion volume in TBI rats.

### 5.3 Neonatal hypoxic-ischemic damage and MSC-EVs

**Figure 4:**
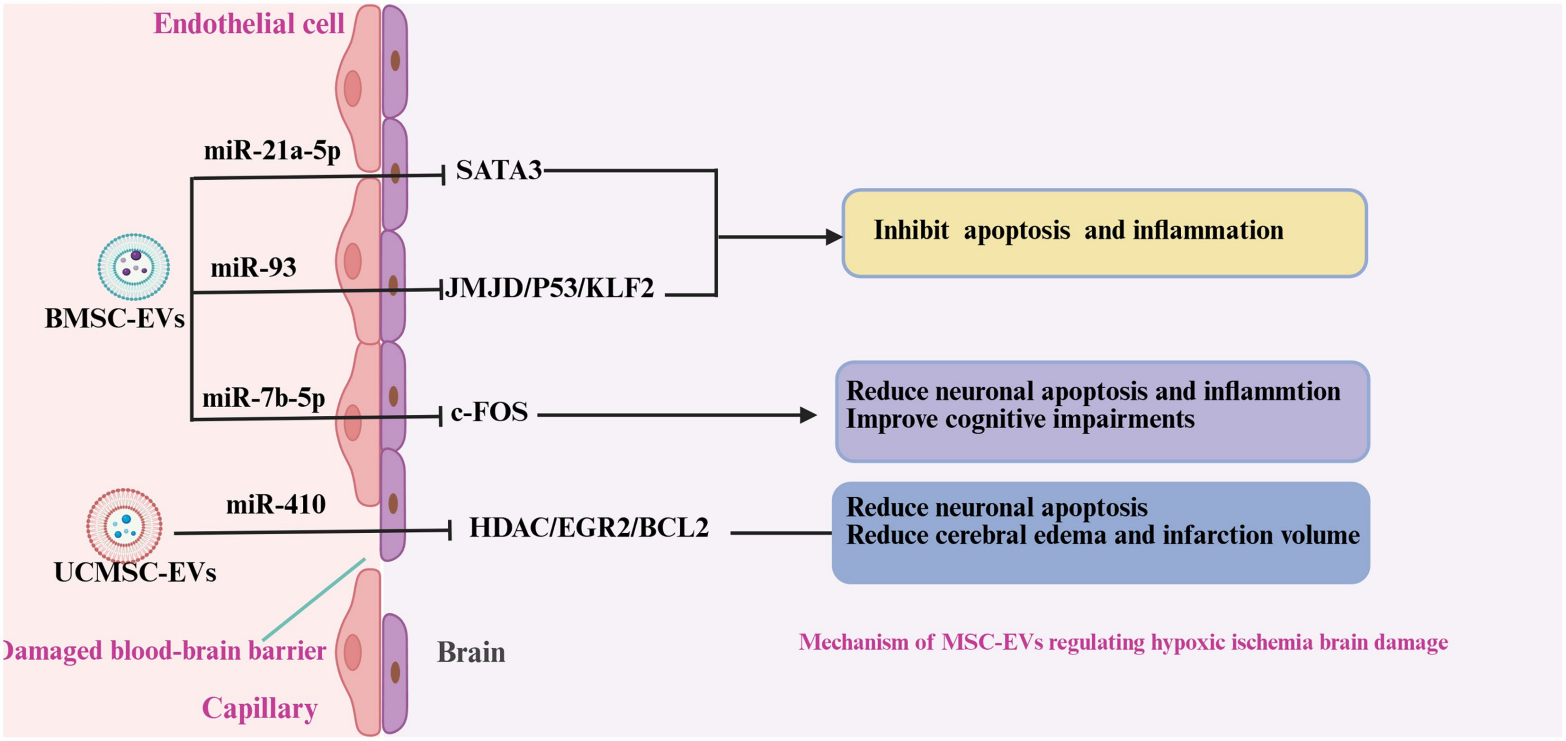
Mechanism of MSC-EVs regulating neonatal hypoxic-ischemic damage. In neonatal ischemic-hypoxic brain injury, BMSC-EVs and UCMSC-EVs released various miRNAs to attenuate the neuroinflammation, neuronal apoptosis, and promote the BBB. Reduced microglia polarity, inhibited inflammation and apoptosis, promoted neuronal proliferation, and reduced brain edema.

Neonatal hypoxic-ischemic damage (HIBD) is a serious neurological disorder caused by perinatal asphyxia, characterized by partial or complete deprivation of cerebral oxygen supply and blood flow during the perinatal period (31, 96). Current clinical management is limited to supportive care, highlighting the urgent need for effective therapeutic interventions. Emerging evidence suggests that MSC-EVs may exert neuroprotective effects by modulating neuroinflammatory responses and improving neurological outcomes in patients with neonatal HIBD (Figure 4).

BMSC-EVs attenuate pro-inflammatory cytokine release, upregulate neurotrophic factors (e.g., BDNF, VEGF, and EGF), and enhance neuronal and vascular endothelial cell proliferation in the subventricular area (44). These neuroprotective effects are mediated by specific miRNAs such as miR-21a-5p and miR-93, which modulate the SATA3 and JMJD/P53/KLF2 signaling pathways, respectively (48, 97). In a hypoxia-ischemia injury model, intracardiac BMSC-EV administration reduced microglial and macrophage activation, inhibited aberrant neuronal phagocytosis, and restored synaptic integrity (46). A previous study on hypoxia-ischemia injury showed that intracardiac injection of BMSC-EVs reduced microglial and macrophage activity, suppressed microglial phagocytosis in normal neurons, and restored neuronal synapses (49). This anti-inflammatory effect is also associated with the inhibition of P38-MAPK and NF-κB activation by BMSC-EVs (52). In contrast to the above findings, Ophelders et al. (53) revealed that BMSC-EVs did not attenuate neuroinflammation in ischemic-hypoxic fetuses but reduced seizure frequency and duration. To strengthen the neuroprotective function of BMSC-EVs, Chu et al. (47) modified BMSC-EVs with hydrogen sulfide and found that post-modification EVs were more abundant in miR-7b-5p. It suppressed c-Fos expression and inhibited the release of inflammatory factors. Osteopontin (OPN), an extracellular matrix glycoprotein, may exacerbate neuroinflammation following cerebral hemorrhage and ischemic or hypoxic brain injury (98, 99). OPN expression is suppressed by BMSC-EVs, which is accompanied by reduced inflammation (49).

Previous studies have shown that UC-MSC-EVs can reduce inflammation in post-ischemic hypoxic brain injury. *In vitro*, UC-MSC EVs upregulated FOXO 3a expression, attenuated microglial pyroptosis, and promoted proliferation after oxygen-glucose deprivation (100). *In vivo*, the intranasal administration of UC-MSC EVs also suppressed microglial activation and inflammatory factor release to alleviate hypoxic brain injury (46). Han et al. (45) demonstrated that UC-MSC-derived EVs are anti-apoptotic and inhibit inflammation, and reported that these effects were associated with the inhibition of the HDAC/EGR2/Bcl-2 pathway by UC-MSC EVs derived from miR-410.

### 5.4 Cardiac arrest and MSC-EVs

Cardiac arrest (CA) is a critical illness that causes acute death and disability worldwide. The survival rate for in-hospital cardiac arrest discharges has been reported to be 7–26% (101, 102). Most survivors suffer from different extents of neurological deficits due to ischemic-hypoxic brain injury (103, 104). To date, there is a lack of effective drugs to alleviate post-resuscitation brain injury (105).

**Figure 5:**
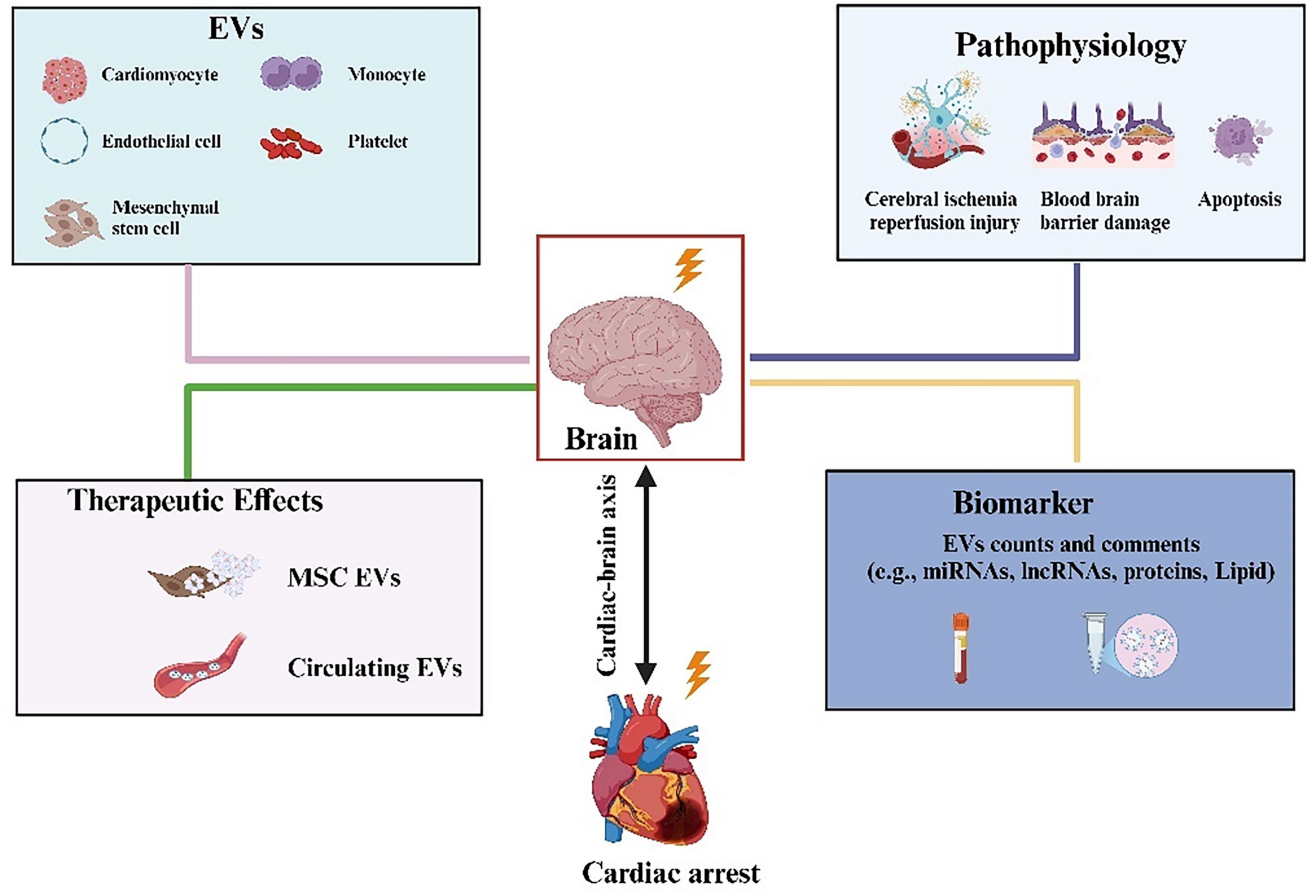
Active participation of EVs from different cellular sources in neuron repair after cardiopulmonary resuscitation. The contents and number of vesicle contents released by various cells, such as erythrocytes, platelets, monocytes, and mesenchymal stem cells, are altered in patients after cardiopulmonary resuscitation. It can be used as a predictor to assess the neurological prognosis of patients after resuscitation.

Although there are few studies on EVs for cardiopulmonary resuscitation, several reports have indicated that EVs play a crucial role in post-resuscitation brain injury. Empana et al. (106) and Sinning et al. (107) found a significant increase in the number of monocyte-and endothelial cell-derived EVs after cardiac arrest. Among patients with STEMI, plasma vesicles were significantly larger in diameter and had elevated levels of GP IIb and PLP-1 in those who experienced out-of-hospital cardiac arrest (OHCA) (108). Based on these findings, Fink et al. (109) detected the expression of three different cell-derived EVs in resuscitated patients. Monocyte- and endothelial-derived EVs were significantly elevated in resuscitated patients, whereas platelet-derived EVs were maintained at normal levels (Figure 5). Among these EVs, monocyte-derived EVs are novel predictors of 20-day survival. Furthermore, a previous study on plasma EVs RNA expression in cardiac arrest/cardiopulmonary resuscitation patients identified that 5,231 lncRNAs and 706 miRNAs were significantly altered (Figure 5). These lncRNAs and miRNAs are mainly responsible for cytokine receptors, cholinergic synapses, mitochondrial respiratory chains, ion channels, and apoptosis (110). Shi et al. (54) reported that BMSC-EVs improve spatial learning and memory capacity in resuscitated rats. This is primarily attributable to the inhibition of neuroinflammation and apoptosis, which promote neurogenesis and angiogenesis. Further studies have shown that this anti-apoptotic and neuroprotective function is relevant to BMSC-EVs derived miR-133b, which regulates the JAK1/AKT/GSK-3β/WNT pathway (85).

## 6 Discussion

### 6.1 Ambiguities in the dosage and criterion for the treatment of ABI with MSC-EVs

**Figure 6:**
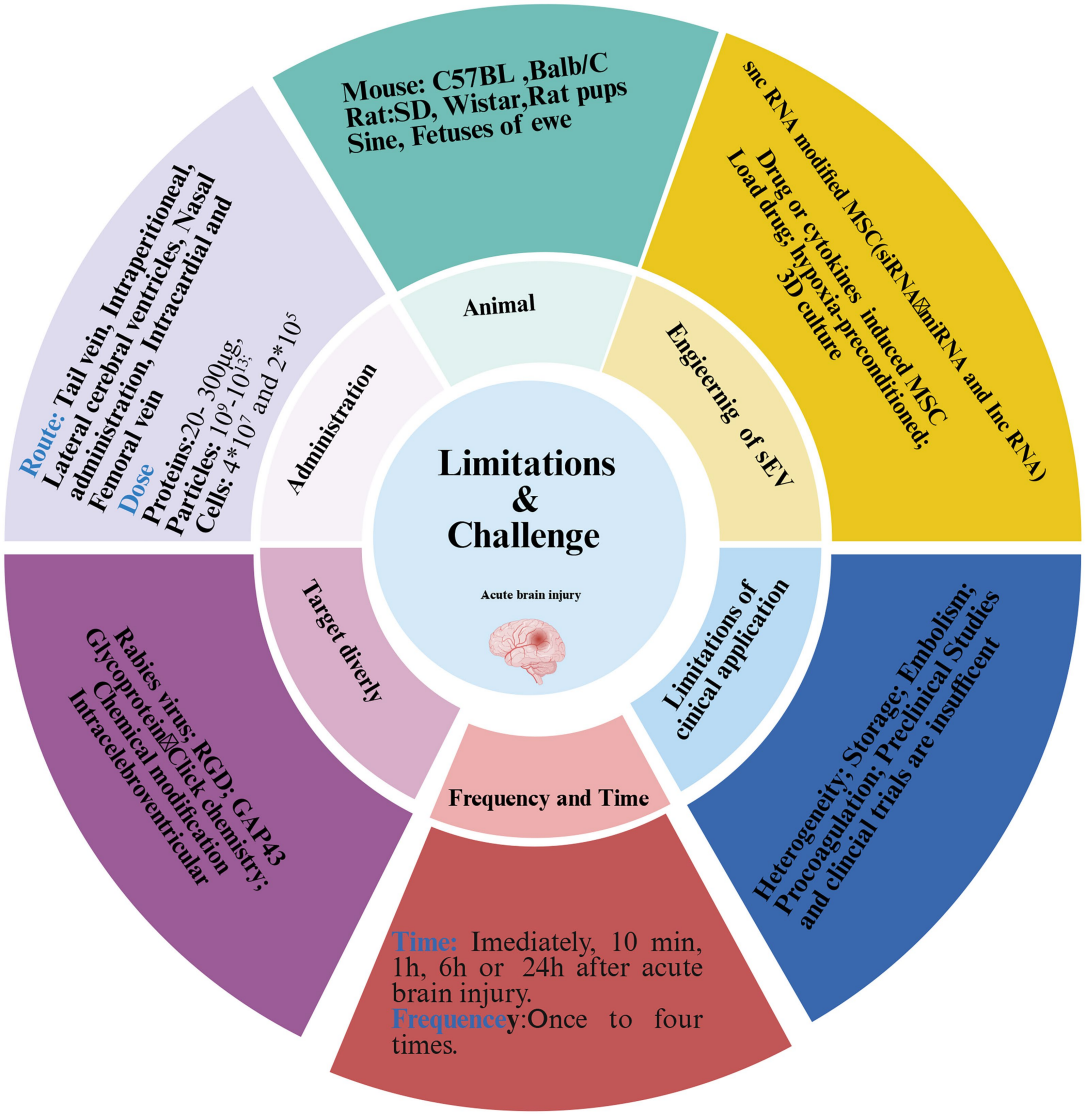
Limitations and challenges of EVs in ABI treatment. Despite many animal model studies demonstrating that MSC-EVs attenuate acute brain injury, there was significant variability in these studies, including animal species, models, dosage, route, and frequency. The heterogeneity, mass production, and storage of MSC-EVs remained to be overcome, and targeting brain transport was also difficult. Furthermore, the side effects of BMSC-EVs have rarely been reported, and preclinical studies are insufficient.

Despite extensive evidence supporting the therapeutics potential of MSC-EVs for ABI, their clinical translation remains limited due to inconsistent preclinical outcomes and a lack of standardized protocols. Substantial ambiguity persists in key experimental parameters, including animal models, dosage, route of administration, and frequency. First, critical methodological inconsistencies existed across studies, particularly in dosing strategies that frequently neglected injury heterogeneity, species differences, animal body mass, and administration routes (111). Second, experimental models range from rodents (mice and rats) to large mammals (pigs and sheep), while delivery approaches (including intravenous, intraperitoneal, intracardiac, intracerebroventricular, and intranasal) differ considerably in both frequency (single to quadruple administration) and dosimetry criteria (Figure 6). The latter manifests as divergent metrics. Most studies employed total protein quantification, whereas others referenced particle counts or cellular equivalence (Figure 6). These discrepancies lead to significant variability in therapeutic outcomes, even within identical ABI models. For instance, reported doses of intravenous BMSC-EVs in murine MCAO models range from 200 to 300 μg (24, 112). A dose–response study on MSC-EVs therapy for TBI found that 100 μg was more effective than 200 μg in promoting angiogenesis and improving neurological deficits (41). *In vitro* models of ABI further suggest an optimal MSC-EVs dose range of 40–50 μg/mL (Supplementary Table S3) (34, 41, 84, 113–116). Due to variations in models, species, and administration time, we recommend that further studies systematically evaluate concentration gradients and time gradients when applying EV-base therapies for ABI. Third, it is important to note that ABI, especially ischemic stroke, often occurs in elderly patients with comorbidities such as hypertension. However, most current ABI models are established in young, healthy rodents without underlying conditions, which limits their clinical relevance. Therefore, we recommend that future relevant studies refer to research on MSC-EVs therapy for stroke and Alzheimer’s disease by using aged or diabetic mice (117, 118). Finally, as described previously, although tail vein injection was the predominant route of administration, phagocytosis by macrophages and differences in tissue distribution in the bloodstream greatly reduced the bioavailability of BMSC-EVs (64, 65).

According to their findings, a single intravenous dose of 100 mg is reasonable for EVs in the treatment of ABI (119). And intracerebroventricular injection may be the optimal route of administration to increase the concentration of EVs in brain tissue (120). Furtherly, we also recommend that both nanoparticle tracking analysis and total protein be quantified in prospective studies of MSC-EVs therapy for ABI in accordance with MISEV guidelines. This may facilitate comparisons of efficacy between different studies.

### 6.2 Safety and potential adverse effects of MSC-EVs

In addition to the lack of standardized dosages and administration protocols, the safety profiles and potential adverse effects of EVs are often neglected by researchers. Research on MSC-EVs for ABI has often followed highly similar pathways, lacking objective evaluation criteria, with a tendency to overemphasize therapeutic benefits while overlooking potential complications. Previous studies reported that EVs may facilitate carcinogenesis under certain conditions (121, 122). For instance, lung macrophages exposed to asbestos release EVs that induce epithelial-mesenchymal transition in pulmonary interstitial cell (123). This pro-oncogenic effect may be attributable to EV-carried miRNAs, arsenic-induced EVs from hepatic epithelial cells, for example, deliver miR-155-5p, activating NF-κB and creating a tumor-favorable inflammatory microenvironment (124).

Beyond carcinogenicity, other documented risks include off-target effects, immune activation, genotoxicity, and thrombotic complications (125). Although the high target specificity of natural EVs may reduce the likelihood of off-target toxicity. This remains a common concern in therapeutic application (125). Some studies may attempt to enhance efficacy by increasing intracranial EV concentrations through higher dose, but this raises the risk of immune reactions and thrombosis. EVs exhibit a tendency to aggregate due to poor zeta potential, which can trigger immune responses (126, 127). MSC-EVs carry proteins such as tetraspanins, integrins, and MHC-I, which are recognized by immune cells. Furthermore, bacterial endotoxins contamination in EV preparations could lead to septic complication. The immunogenicity of MSC-EVs depends on factors including the differentiation state of the parent cells, vesicle size, cargo composition, storage conditions, and infusion rate (128). EVs derived from highly differentiated or large parental cells are particularly prone to inducing immune response (128). Several reports have found that MSC-EVs influence coagulation pathways (129–131). ADMSC-EVs shorten clotting time via both the extrinsic and intrinsic pathway (129). Similarly, UC-MSC-EVs also promote coagulation process in a dose-and tissue factor-dependent manner (130). This effect may be mediated by TF expression on EVs, which enhances FXa production and accelerates clot formation (131). Pre-treatment with heparin has been shown to mitigate EV-induced thrombosis and reduce pulmonary embolism risk *in vivo* (130).

### 6.3 Major challenges in the clinical translation

Despite the considerable therapeutic potential of MSC-EVs, their clinical translation remains protracted. Analysis of trial registries^1^ indicated predominant applications for COVID-19, ARDS, and metabolic diseases, with ABI representing a minority indication (119, 132). This delayed translation reflects multidimensional challenges wherein production standardization constitutes a primary bottleneck: heterogeneous isolation techniques, including ultracentrifugation, microfluidics, and immunocapture, yield preparations with significant variations in size, cargo composition (e.g., miRNAs/proteins), and functional reproducibility (132). Safety assessment remains paramount. Maximizing the purity of MSC-EVs by minimizing manufacturing-derived impurities is essential (133). *In vitro* toxicity studies indicate that MSC-EVs were free from bacterial endotoxins, and show no genotoxic, hemolytic, platelet-aggregating or complement-activating properties. However, high doses can promote leukocyte proliferation. In contrast, bovine milk-derived EVs have been shown to contain endotoxins capable of inducing hemolysis, platelet aggregation, and complement activation, with adverse effects intensifying at higher concentrations (134). It is estimated that systemic EV therapy in humans may require approximately one trillion MSC-EVs per administration (135).

### 6.4 Critical perspective

To alleviate neurological impairment following ABI, numerous strategies have been explored, including antioxidants or NMDA receptor antagonists aimed at mitigating neuroinflammation (5, 136). However, these conventional neuroprotective agents are limited by single-target specificity, poor BBB penetration, and significant side effects, rendering them inadequate against the multifaceted pathology of ABI (137). In contrast, MSC-EVs serve as natural nanocarriers with excellent biocompatibility and high BBB permeability (13). They enable multi-mechanism regulation through the delivery of diverse bioactive molecules (e.g., miRNAs, proteins), suppressing neuroinflammation, reducing oxidative stress, promoting angiogenesis, and facilitating synaptic remodeling. Moreover, MSC-EVs can be engineered to enhance targeting and therapeutic efficacy, overcoming key limitations of conventional drugs (17). Surface modifications with targeting ligands, such as (arginine-glycine-aspartate) RGD peptides, which bind integrins on endothelial cells, can improve uptake and delivery (138–140). For example, RGD-C1C2-modified ReN cell-derived EVs facilitate targeted intracranial delivery and enhance anti-inflammatory effects in MCAO mice (34). The rabies virus glycoprotein (RVG), a neuron-specific viral peptide, has been employed to generate neuron-targeted delivery. Yang et al. (141) developed RVG-LAMP-modified BMSC-EVs that achieved successful brain-targeted delivery. Recent approaches also include click chemistry and metabolic labeling for attaching functional groups or therapeutic molecules to EV surfaces (63). Thus, MSC-EVs represent an innovative and comprehensive neurorepair strategy, offering the potential to overcome the efficacy barriers of conventional neuroprotection (14).

Despite promising advances in the use of MSC-EVs for ABI therapy, several challenges must be addressed to enable clinical translation. First, there is an urgent need to establish standardized preclinical frameworks, including, animal models, genetic backgrounds, administration routes (e.g., intravenous vs. intracerebroventricular), dosing metrics (particle count/protein mass), treatment frequency, and functional endpoints, to enable cross-study comparability. Second, scaling production under Good Manufacturing Practice (GMP)-compliant condition remains a major hurdle. Innovative isolation platforms and strict quality control, including purity, potency and reproducibility, are essential (138, 142). MSC-EVs products must comply with Food and Drug Administration (FDA) guidelines, requiring full disclosure of chemical, manufacturing, and control (CMC) information (135). Third, while MSC-EVs possess innate homing capabilities, their targeting efficiency remains suboptimal within the complex milieu of neuropathological injury. Engineering strategies are therefore essential to improve cell-type specificity and delivery precision (143). Forth, the inherent heterogeneity of BMSC-EV preparations must be addressed through genetic or pharmacological preconditioning approaches, which enhance therapeutic efficacy by modulating bioactive cargo (22, 144). For example, BDNF-overexpressing HEK293-derived EVs conferred 3.2-fold greater neuroprotection against ischemia-reperfusion-induced neural apoptosis compared with unmodified EVs (145). Fifth, comprehensive toxicological profiling of MSC-EVs is imperative, particularly concerning procoagulant tendencies and immunogenic reactions. Strategies to mitigate these risks include reducing injection frequency, employing genetic editing to downregulate MHC-I expression, pretreating with heparin, and administering infusions at slower rates to minimize coagulation activation (125). It is also critical to recognize that EVs derived from diverse cellular sources—such as endothelial cells, microglia, astrocytes, and MSCs—exhibit distinct functional profiles and collectively contribute to the pathophysiology of ABI (114, 146). Most prior studies have focused exclusively on a single EV type, overlooking this complex intercellular communication.

Looking ahead, engineering modifications represent a promising avenue for enhancing the neuroprotective effects of MSC-EVs and constitute a major future direction for the field. Robust clinical evaluation will require large-scale, multicenter collaborative efforts (147). Close collaboration among researchers, regulators, clinicians and industry partners is crucial for accelerating the translation and commercialization of MSC-EV-based therapies. Engagement with patient advocacy groups and other stakeholders will further ensure that development is ethical, equitable, and focused on patient accessibility and affordability (148). By addressing these challenges through shared standards and collaborative science, MSC-EV therapies may soon offer safe, effective, and accessible treatments for patients with ABI. Standardized protocols, best practices, and open knowledge exchange will be vital to fully realize the potential of this promising therapeutic approach (148). With technological advances and better understanding, MSC-EVs are expected to become an attractive therapeutic option for alleviating ABI and improving neurological prognosis.

## Footnotes

1
https://clinicaltrials.gov
