# Supplementary material for: Beyond the hype: re-evaluating efficacy metrics and modeling rigor for MSC-EVs-based therapy in acute brain injury
Source: Front Med (Lausanne). 2025 Dec 4;12:1654429. doi: 10.3389/fmed.2025.1654429 (PMC12711728; doi:10.3389/fmed.2025.1654429)
Supplement: Supplementary file 1 [file Table_1.docx]

**TABLE S1**. MSC-EVs alleviated TBI in vivo.

| Species | Cells | Administration route | Time | Dose of EVs | Ref. |
| --- | --- | --- | --- | --- | --- |
| Wistar rat | BMSC | Tail vein | 1 day after TBI | 100 μg | (35) |
| SD rat | BMSC | Tail vein | 1 h after CCI | 100 μg | (36) |
| C57BL | BMSC | Tail vein | After TBI | 1.26×10^9^ | (37) |
| SD rat | BMSC | Femoral vein | After surgery | 200 μg | (38) |
| SD rat | BMSC | Tail vein | 1 h post bleeding | 100 μg | (39) |
| Swine | BMSC | Vein | 6 h post TBI | 1×10^13^ | (40) |
| Wistar rat | BMSC | Tail vein | 1 day after TBI | 50, 100 and 200 μg/ml | (41) |
| Wistar rat | BMSC | Tail vein | 24 h after TBI | 100 μg | (42) |
| SD rat | BMSC | Tail vein | 24 h after TBI | 100 μg | (43) |
| SD rat | ADMSC | Lateral ventricle | 24 h after injury | 20 μg | (44) |

Abbreviations: ADMSC, adipose mesenchymal stem cell; BMSC, bone marrow mesenchymal stem cell; CCI, controlled cortical impact injury; TBI, traumatic brain injury; UC-MSC, umbilical cord mesenchymal stem cell.
