# Supplementary material for: Beyond the hype: re-evaluating efficacy metrics and modeling rigor for MSC-EVs-based therapy in acute brain injury
Source: Front Med (Lausanne). 2025 Dec 4;12:1654429. doi: 10.3389/fmed.2025.1654429 (PMC12711728; doi:10.3389/fmed.2025.1654429)
Supplement: Supplementary file 2 [file Table_2.docx]

**TABLE S2**. MSC-EVs alleviated HIBD and CA in vivo.

| Model | Species | Cells | Administration route | Time | Dose of EVs | Ref. |
| --- | --- | --- | --- | --- | --- | --- |
| HIBD | Neonatal mouse | UC-MSC | Intraperitoneal | First: 14 h before HI; Second: before HI；  Third: immediately after HI;  Fourth: 3 h after HI. | Released by 2×10^5^ MSCs | (45) |
|  | 2-day-old Wistar | UC-MSC | Nasal | Between the LPS injection and the ligation | 50 mg/kg | (46) |
|  | Postnatal day 7–10 C57BL | BMSC | Intracardial | 24 h after HI | 100 μg | (47) |
|  | 7-day-old neonatal mice | BMSC | Nasal | After HI | 2 × 10^9^ | (48) |
|  | 7-day-old neonatal mice | BMSC | Intracardial | At 24 h after HI insult | 100 μg | (49) |
|  | Postnatal day 9 C57BL | BMSC | Nasal | After HI | 1.25×10^9^ | (50) |
|  | Postnatal day 9-12 C57BL | BMSC | Intraperitoneally | At 24, 72, and 120 h post-HI | 10 μl/g | (51) |
|  | 2-day SD rat pulp | BMSC | Lateral ventricle | After HI | NA | (52) |
|  | Fetuses of ewes | BMSC | Vein | 1 h after surgery; 4 days after the HI | Released by 4×10^7^ MSCs | (53) |
| CA | SD rat | BMSC | Lateral ventricles | 24 h before DHCA | 30 μg | (54) |

Abbreviations: BMSC, bone marrow mesenchymal stem cell; CA, cardiac arrest; DHCA, hypothermic circulatory arrest; HI, Hypoxic injury; HIBD, hypoxic ischemic brain damage; UC-MSC, umbilical cord mesenchymal stem cell
