# Supplementary material for: Beyond the hype: re-evaluating efficacy metrics and modeling rigor for MSC-EVs-based therapy in acute brain injury
Source: Front Med (Lausanne). 2025 Dec 4;12:1654429. doi: 10.3389/fmed.2025.1654429 (PMC12711728; doi:10.3389/fmed.2025.1654429)
Supplement: Supplementary file 3 [file Table_3.docx]

**Table S3.** Studies on the dose-effect relationship of EVs in the treatment of ABI.

| Cell source | Dose | Models | Species/Cells | Administration  Route | Administration time | Evaluate time | Indicators | Optimal concentration | Ref. |
| --- | --- | --- | --- | --- | --- | --- | --- | --- | --- |
| BMSC | 50 μg, 100 μg and 200 μg | TBI | Wistar rat | Tail vein | At 24 h, 4^th^ and 7^th^ day after TBI | On day 35 after TBI | mNSS, Sensorimotor and cognitive function, angiogenesis, neurogenesis and neuroinflammation. | 100 μg. | (41) |
| BMSC | 2🞩10^6^ MSC Eq/kg and 2 🞩10^7^ MSC Eq/kg | MCAO | SD rat | Tail vein | At 24 h, 3 days, 7 days and 14 days post MCAO | On day 28 after MCAO | Infarct volume, body weight, macrophage infiltrate, microglia accumulation, and angiogenesis | No difference | (114) |
| Astrocyte | 10， 20 and 40 μg/ml | MCAO | Wistar rat | Tail vein | Reperfusion | At 24 h reperfusion | Neurological deficit scores, infarct volume, brain water content, neuroinflammation. | 20 ug/ml | (115) |
| BMSC | 20 μg/ml and 40 μg/ml | OGD/R | Primary neuron | In vitro | Reoxygenation | 24 hours at 37 °C as reoxygenation | TUNEL assays and Wester blot (Cleaved-caspase 3 and Bax). | 40 ug/ml | (84) |
| Astrocyte | 5, 10，20，40 μg/ml | OGD | Primary neuron | In vitro | OGD | OGD | Cell viability was detected by CCK8 assays. | 20 μg/ml | (116) |
| BMSC | 0.1,1,10,25,50, 100, 250 μg/ml | Normal | Endothelial cell | In vitro | - | Physiological conditions | Cell viability, migration, and  tube formation | 50 μg/ml | (117) |
| NPC | 10, 20 and 40 μg/ml | LPS treatment | BV2 | In vitro | - | 24 h after LPS treatment | Inflammation factor | 40 ug/ml | (118) |

Abbreviations: BMSC, bone marrow mesenchymal stem cell; LPS, lipopolysaccharide; MCAO, middle cerebral artery occlusion; NSC, neural stem cell ;OGD, oxygen glucose deprivation; OGD/R, oxygen glucose deprivation/reoxygenation; TBI, traumatic brain injury; TUNEL, TdT-mediated dUTP nick end labeling.
